# Accuracy and economic evaluation of screening tests for undiagnosed COPD among hypertensive individuals in Brazil

**DOI:** 10.1038/s41533-022-00303-w

**Published:** 2022-12-13

**Authors:** S. M. Martins, A. P. Dickens, W. Salibe-Filho, A. A. Albuquerque Neto, P. Adab, A. Enocson, B. G. Cooper, L. V. A. Sousa, A. J. Sitch, S. Jowett, R. Adams, K. K. Cheng, C. Chi, J. Correia-de-Sousa, A. Farley, N. Gale, K. Jolly, M. Maglakelidze, T. Maghlakelidze, K. Stavrikj, A. M. Turner, S. Williams, R. E. Jordan, R. Stelmach

**Affiliations:** 1 Family Medicine, ABC Medical School, Sao Paulo, Brazil; 2 Institute of Applied Health Research, University of Birmingham, Birmingham, UK; 3 Observational and Pragmatic Research Institute, Midview City, Singapore; 4 Respiratory Division, Hospital das Clínicas, Faculdade de Medicina, Universidade de Sao Paulo, Sao Paulo, Brazil; 5 Faculty of Medicine, Federal University of São Paulo, Sao Paulo, Brazil; 6 Lung Function & Sleep, University Hospitals Birmingham NHS Foundation Trust, Birmingham, UK; 7 NIHR Birmingham Biomedical Research Centre, University Hospitals Birmingham NHS Foundation Trust and University of Birmingham, Birmingham, UK; 8 Department of General Practice, Peking University First Hospital, Beijing, China; 9 International Primary Care Respiratory Group, Edinburgh, UK; 10 Life and Health Sciences Research Institute (ICVS), School of Medicine, University of Minho, Braga Portugal, ICVS/3B’s, PT Government Associate Laboratory, Braga/Guimarães, Portugal; 11 Health Services Management Centre, School of Social Policy, College of Social Sciences, University of Birmingham, Birmingham, UK; 12 Georgian Respiratory Association, Tbilisi, Georgia; 13 Petre Shotadze Tbilisi Medical Academy, Tblisi, Georgia; 14 Ivane Javakhishvili Tbilisi State University, Tblisi, Georgia; 15 Center for Family Medicine, Faculty of Medicine, Ss.Cyril and Methodius University in Skopje, Skopje, North Macedonia

**Keywords:** Chronic obstructive pulmonary disease, Diagnosis

## Abstract

In Brazil, prevalence of diagnosed COPD among adults aged 40 years and over is 16% although over 70% of cases remain undiagnosed. Hypertension is common and well-recorded in primary care, and frequently co-exists with COPD because of common causes such as tobacco smoking, therefore we conducted a cross-sectional screening test accuracy study in nine Basic Health Units in Brazil, among hypertensive patients aged ≥40 years to identify the optimum screening test/combinations to detect undiagnosed COPD. We compared six index tests (four screening questionnaires, microspirometer and peak flow) against the reference test defined as those below the lower limit of normal (LLN-GLI) on quality diagnostic spirometry, with confirmed COPD at clinical review. Of 1162 participants, 6.8% (*n* = 79) had clinically confirmed COPD. Peak flow had a higher specificity but lower sensitivity than microspirometry (sensitivity 44.3% [95% CI 33.1, 55.9], specificity 95.5% [95% CI 94.1, 96.6]). SBQ performed well compared to the other questionnaires (sensitivity 75.9% [95% CI 65.0, 84.9], specificity 59.2% [95% CI 56.2, 62.1]). A strategy requiring both SBQ and peak flow to be positive yielded sensitivity of 39.2% (95% CI 28.4, 50.9) and specificity of 97.0% (95% CI 95.7, 97.9). The use of simple screening tests was feasible within the Brazilian primary care setting. The combination of SBQ and peak flow appeared most efficient, when considering performance of the test, cost and ease of use (costing £1690 (5554 R$) with 26.7 cases detected per 1,000 patients). However, the choice of screening tests depends on the clinical setting and availability of resources.

ISRCTN registration number: 11377960.

## Introduction

Chronic obstructive pulmonary disease (COPD) represents a major challenge to global health due to its increasing incidence and mortality, and it is currently the third leading cause of death worldwide^1^. The high social and health costs associated with COPD are attributable largely to hospitalization and productivity loss from exacerbations, estimated to be €48.4 billion per year for Europe in 2013^2^ and high costs are also seen in Brazil. A systematic review of economic studies suggested that earlier identification of COPD and reducing exacerbations would be key drivers for reducing costs^3^. COPD has a gradual onset over a number of years^4^ and most COPD cases are identified during an exacerbation or after significant loss of lung function^5^. Timely diagnosis of COPD remains limited worldwide and especially in lower income countries^6^, due to various factors including patients not recognizing or adapting to their symptoms^7^, but also due to lack of clinical expertize and unavailability of spirometry in primary care^8,9^.

The prevalence of COPD in Sao Paulo city in individuals over 40 years old is estimated to be 15.8%^10^, but initial data suggest over 70% of cases are undiagnosed^11^, similar to many other countries worldwide^12^. Primary care is the ideal setting for implementing programmes for earlier identification of COPD, potentially offering relatively straightforward access to and identification of symptomatic patients with clinically significant disease who will benefit from currently available therapeutic interventions^13^.

The diagnosis of COPD is based on demonstration of airflow obstruction on spirometry in those with chronic respiratory symptoms, who have risk factors for the disease^14,15^. Diagnostic spirometry, the gold standard confirmatory test for COPD, is underused in primary health-care in Brazil^16^. Barriers to its use include poor access to a spirometer, the time-consuming nature of pre- and post-bronchodilator spirometry within limited consultation appointments and, lack of experience and unfamiliarity with how to perform and interpret the exam^16^. Given these difficulties, use of screening tests that are more feasible in primary care could optimize referrals for specialist diagnostic spirometry; referring only those most likely to have disease. While general population screening programmes are not currently recommended in asymptomatic patients^17,18^ earlier detection of undiagnosed but clinically significant COPD could enable appropriate treatments and the modification of risk factors such as smoking, which may improve patient health outcomes^19^ and potentially result in cost savings^20^.

Several screening tests have been developed to identify undiagnosed COPD, including questionnaires and airflow measurement devices^21–25^. However, there is no consensus on the optimal test or combination of tests to use^26^, and this may depend on the setting and resources available. Previous screening test accuracy studies were conducted in high-income countries, where smoking is the main risk factor for COPD; it is unknown whether test performance is similar in countries such as Brazil, where the distribution of risk factors and the severity of undiagnosed disease may differ from higher income settings.

The purpose of our study was to assess the accuracy and associated costs of selected individual screening tests and their combinations for detecting undiagnosed COPD in a primary care setting. To maximize detecting undiagnosed patients who had most to benefit from earlier diagnosis, we targeted patients known to have systemic arterial hypertension (defined by being on a primary care hypertension register); hereafter referred to as hypertension. Hypertension is well-recorded in Brazil, and is a common comorbidity among patients with COPD due to shared risk factors such as cigarette smoking. This approach offered a feasible way to identify high rates of undiagnosed COPD.

## Results

### Sample

**Fig. 1:**
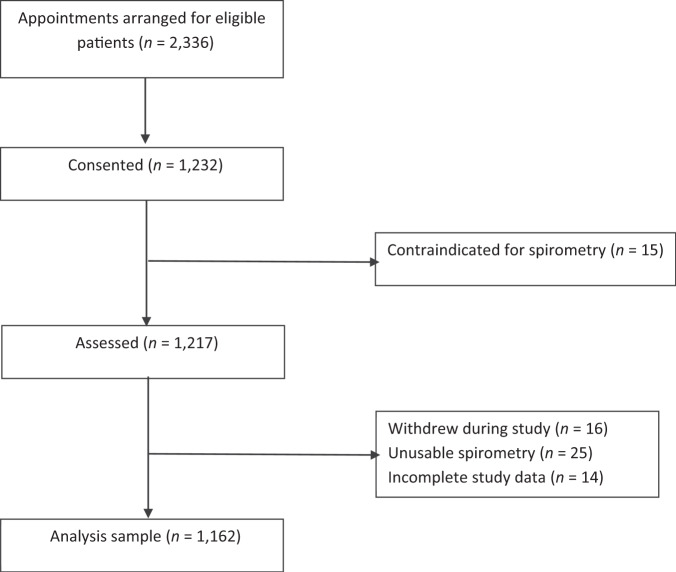
Recruitment flowchart.

We invited 2236 individuals with diagnosed hypertension and 1232 (55.1%) consented to participate in the study. Of these, 15 had contraindications for spirometry hence 1217 (54.4%) were assessed (Fig. 1). During the study, 16/1217 (1.3%) participants withdrew, 25/1217 (2.1%) had unusable spirometry and 14/1217 (1.2%) did not have complete index and reference test data, leaving an analysis sample of 1162 individuals.

The mean age of study participants was 62.3 (SD 10.1) years, 378 (32.5%) were men, 682 (58.7%) reported white ethnicity and the majority (*n* = 988, 85.0%) lived in urban areas. Half of the participants (*n* = 592, 50.9%) had a positive smoking history of whom 146 were current smokers, 944 (81.2%) reported no, or mild breathlessness (mMRC score 0–1) and 114 (9.8%) reported a prior diagnosis of COPD, chronic bronchitis or emphysema (Table 1).

**Table 1:** Characteristics of study participants.

Characteristic	Total sample (*n* = 1162)	Reference test positive (*n* = 79)	Reference test negative (*n* = 1083)
Sex; *n* (%) male	378 (32.5)	39 (49.4)	339 (31.3)
Age; mean (SD)	62.3 (10.1)	66.4 (10.8)	62.0 (9.9)
Ethnicity; *n* (%)			
White	682 (58.7)	42 (53.2)	640 (59.1)
Black	253 (21.8)	19 (24.1)	234 (21.6)
Asian	56 (4.8)	5 (6.3)	51 (4.7)
Other	171 (14.7)	13 (16.5)	158 (14.6)
BMI; mean (SD)	30.6 (5.7)	29.0 (6.2)	30.7 (5.7)
Education; *n* (%)			
Illiterate	161 (13.9)	20 (25.3)	141 (13.0)
Elementary school I or II	625 (53.8)	45 (57.0)	580 (53.6)
High school	322 (27.7)	14 (17.7)	308 (28.4)
Degree or above	54 (4.7)	0 (0)	54 (5.0)
Employment status; *n* (%)			
Work and Study	18 (1.6)	0 (0)	18 (1.7)
Work only	320 (27.65)	16 (20.3)	304 (28.1)
On leave or incapacitated to work	15 (1.3)	1 (1.3)	14 (1.3)
Retired	531 (45.7)	46 (58.2)	485 (44.8)
Neither working or studying	277 (23.8)	16 (20.3)	261 (24.1)
Missing	1 (0.1)	0 (0)	1 (0.1)
Living area; *n* (%)			
Urban	988 (85.0)	67 (84.8)	921 (85.0)
Rural	172 (14.8)	12 (15.2)	160 (14.8)
Missing	2 (0.2)	0 (0)	2 (0.2)
Smoking status; *n* (%)			
Current smoker	146 (12.6)	21 (26.6)	125 (11.5)
Ex-smoker	446 (38.4)	48 (60.8)	398 (36.8)
Never smoker	570 (49.1)	10 (12.7)	560 (51.7)
Pack years; mean (SD)	12.4 (22.3)	32.5 (32.7)	10.9 (20.6)
Previously diagnosed conditions; n (%)			
COPD/chronic bronchitis/emphysema	114 (9.8)	27 (34.2)	87 (8.0)
Asthma	103 (8.9)	18 (22.8)	85 (7.9)
Tuberculosis	30 (2.6)	4 (5.1)	26 (2.4)
Diabetes mellitus	334 (28.7)	13 (16.5)	321 (29.6)
Anxiety	387 (33.3)	22 (27.9)	365 (33.7)
Depression	208 (17.9)	8 (10.1)	200 (18.5)
Heart disease	201 (17.30)	15 (19.0)	186 (17.2)
Cancer	58 (5.0)	4 (5.1)	54 (5.0)
None of the above	349 (30.0)	20 (25.3)	329 (30.4)
Respiratory symptoms, *n* (%)			
At least occasional wheeze	390 (33.6)	45 (57.0)	345 (31.9)
Productive cough	316 (27.2)	35 (44.3)	281 (26.0)
mMRC; *n* (%)			
Grade 0–1	944 (81.2)	55 (69.6)	889 (82.1)
Grade 2–4	218 (18.8)	24 (30.4)	194 (17.9)
CAT; mean (SD)	9.0 (7.9)	12.7 (8.6)	8.8 (7.7)
Exposure to pollutants^a^; *n* (%)			
Current/past exposure	1056 (90.9)	72 (91.1)	984 (90.9)
Never	105 (9.0)	7 (8.9)	99 (9.1)
Missing	1 (0.1)	0 (0)	1 (0.1)
GOLD stage if reference test positive; *n* (%)			
I (FEV_1_ ≥ 80% predicted)	—	19 (24.1)	—
II (FEV_1_ 50–79% predicted)	—	45 (57.0)	—
III (FEV_1_ 30–49% predicted)	—	14 (17.7)	—
IV (FEV_1_ < 30% predicted)	—	1 (1.3)	—

^a^Chemicals, particulates, cooking fumes, biomass fuel, steams, gas, dust.

**Fig. 2:**
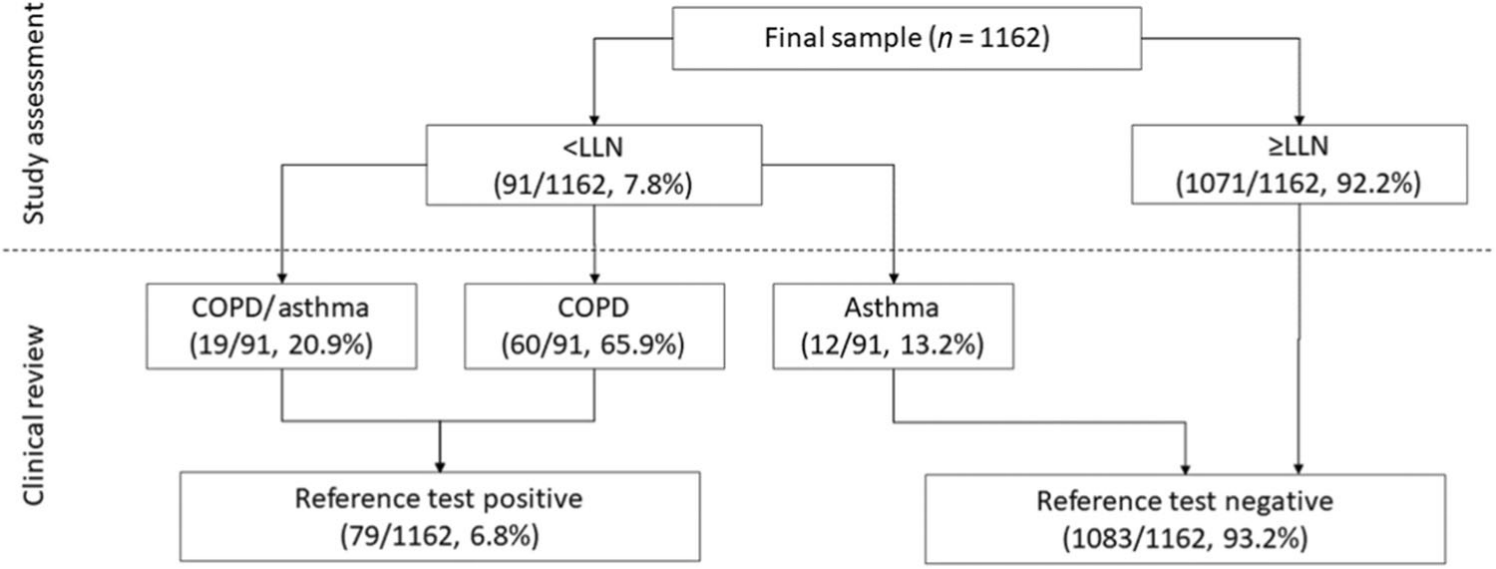
Reference test outcomes. LLN Lower Limit of Normal.

Over half (*n* = 652, 56.1%) of the participants had good quality spirometry, while data for the remaining 510 (43.9%) was defined as usable. Ninety-one (7.8%) participants had spirometry-confirmed airflow obstruction using the LLN criteria. After clinical review, 12 (13.2%) of these participants were diagnosed with asthma, 19 (20.9%) with asthma/COPD overlap and 60 (65.9%) clinically confirmed COPD; the latter two groups were defined as reference test positive for this study (Fig. 2). Based on study spirometry, prevalence of obstruction was 27.2% (*n* = 31) in participants reporting previous COPD, chronic bronchitis or emphysema diagnoses, and 5.7% (*n* = 60) amongst the remaining sample. According to the FEV_1_/FVC < 0.7 criteria, 11.9% (*n* = 138) of all participants had airflow obstruction. Forty-eight (34.8%) participants were defined as obstructed on the FEV_1_/FVC < 0.7 criteria but not the LLN, of which eight (16.7%) reported a previous COPD diagnosis.

### Performance of individual index tests and test combinations

Among screening questionnaires, COPD-SQ had the highest sensitivity (78.5%; 95% CI 67.8%, 86.9%), followed by SBQ (75.9%; 95% CI 65.0%, 84.9%), while the CDQ had the lowest sensitivity (50.6%; 95% CI 39.1%, 62.1%). Conversely, CDQ had the highest specificity (77.3%; 95% CI 74.7%, 79.7%), with SBQ and COPD-SQ demonstrating specificities of 59.2% (95% CI 56.2%, 62.1%) and 51.2% (95% CI 48.1%, 54.2%), respectively (Table 2). Overall, COPD-SQ and SBQ performed best, if we consider sensitivity and specificity to be equally important.

**Table 2:** Accuracy of index tests and test combinations.

Part 1	Part 2	Strategy type	TP	FP	TN	FN	Sensitivity % (95% CI)	Specificity % (95% CI)	PPV % (95% CI)	NPV % (95% CI)
CAPTURE	n/a	Individual	48	590	493	31	60.8 (49.1; 71.6)	45.5 (42.5; 48.5)	7.5 (5.6; 9.9)	94.1 (91.7; 95.9)
CDQ	n/a	Individual	40	246	837	39	50.6 (39.1; 62.1)	77.3 (74.7; 79.7)	14.0 (10.2; 18.6)	95.5 (94.0; 96.8)
SBQ	n/a	Individual	60	442	641	19	75.9 (65.0; 84.9)	59.2 (56.2; 62.1)	12.0 (9.3; 15.1)	97.1 (95.5; 98.3)
COPD-SQ	n/a	Individual	62	529	554	17	78.5 (67.8; 86.9)	51.2 (48.1; 54.2)	10.5 (8.1; 13.2)	97.0 (95.3; 98.3)
Peak flow	n/a	Individual	35	49	1034	44	44.3 (33.1; 55.9)	95.5 (94.1; 96.6)	41.7 (31.0; 52.9)	95.9 (94.6; 97.0)
Microspirometry	n/a	Individual	40	139	944	39	50.6 (39.1; 62.1)	87.2 (85.0; 89.1)	22.3 (16.5; 29.2)	96.0 (94.6; 97.2)
CAPTURE	Peak flow	Combined	25	31	1052	54	31.6 (21.6; 43.1)	97.1 (96.0; 98.0)	44.6 (31.3; 58.5)	95.1 (93.7; 96.3)
CDQ	Peak flow	Combined	20	25	1058	59	25.3 (16.2; 36.4)	97.7 (96.6; 98.5)	44.4 (29.6; 60.0)	94.7 (93.2; 96.0)
SBQ	Peak flow	Combined	31	33	1050	48	39.2 (28.4; 50.9)	97.0 (95.7; 97.9)	48.4 (35.8; 61.3)	95.6 (94.2; 96.8)
COPD-SQ	Peak flow	Combined	31	36	1047	48	39.2 (28.4; 50.9)	96.7 (95.4; 97.7)	46.3 (34.0; 58.9)	95.6 (94.2; 96.8)
CAPTURE	Microspirometry	Combined	24	77	1006	55	30.4 (20.5; 41.8)	92.9 (91.2; 94.3)	23.8 (15.9; 33.3)	94.8 (93.3; 96.1)
CDQ	Microspirometry	Combined	23	42	1041	56	29.1 (19.4; 40.4)	96.1 (94.8; 97.2)	35.4 (23.9; 48.2)	94.9 (93.4; 96.1)
SBQ	Microspirometry	Combined	32	68	1015	47	40.5 (29.6; 52.1)	93.7 (92.1; 95.1)	32.0 (23.0; 42.1)	95.6 (94.2; 96.7)
COPD-SQ	Microspirometry	Combined	33	72	1011	46	41.8 (30.8; 53.4)	93.4 (91.7; 94.8)	31.4 (22.7; 41.1)	95.6 (94.2; 96.8)

*TP* true positive, *FP* false positive, *TN* true negative, *FN* false negative, *PPV* positive predictive value, *NPV* negative predictive value.

Combined strategy = requires both tests to be positive for screen positivity.

The CDQ had the largest differences with other questionnaires, with significantly lower sensitivity than the SBQ (−25.3%; 95% CI −36.8%, −13.8%; <0.001) and the COPD-SQ (−27.8%; 95% CI −40.2%, −15.5%; <0.001) (Table 3), but with an increase in specificity of between 18.1 percentage points (95% CI 15.4%, 20.8%; <0.001) and 31.8 percentage points (95% CI 28.1%, 35.4%; <0.001) compared to all other questionnaires (Table 4).

**Table 3:** Comparative sensitivity for individual index tests.

Individual test	CDQ		SBQ		COPD-SQ		Peak flow		Microspirometry	
	Difference (95% CI)	*p*	Difference (95% CI)	*p*	Difference (95% CI)	*p*	Difference (95% CI)	*p*	Difference (95% CI)	*p*
Capture	10.1 (−4.5. 24.8)	0.200	−15.2 (−28.1; −2.2)	0.023	−17.7 (−29.94; −5.49)	0.004	16.5 (1.40; 31.50)	0.035	10.1 (−6.7; 26.92)	0.268
CDQ			−25.3 (−36.8; −13.8)	<0.00001	−27.8 (−40.2; −15.5)	<0.00001	6.3 (−9.5; 22.2)	0.500	0 (−15.7; 15.7)	1.000
SBQ					−2.5 (−11.6; 6.6)	0.754	31.6 (17.9; 45.3)	<0.00001	25.3 (10.2; 40.4)	0.001
COPD-SQ							34.1 (20.3; 48.0)	<0.0001	27.8 (13.0; 42.7)	0.001
PEF									−6.3 (−20.9; 8.2)	0.458

Values indicate the difference in sensitivity (with 95% CI & McNemar’s *p*-values), comparing the index test in the left column against index tests across the table.

**Table 4:** Comparative specificity for individual index tests.

Variable	CDQ		SBQ		COPD-SQ		Peak flow		Microspirometry	
	Difference (95% CI)	*p*	Difference (95% CI)	*p*	Difference (95% CI)	*p*	Difference (95% CI)	*p*	Difference (95% CI)	*p*
Capture	−31.8 (−35.4. −28.1)	<0.00001	−13.7 (−17.2; −10.1)	<0.00001	−5.6 (−9.3; −.2.0)	0.002	−50.0 (−53.2; −46.7)	<0.00001	−41.6 (−45.3; −38.0)	<0.00001
CDQ			18.1 (15.4; 20.8)	<0.00001	26.1 (23.0; 29.3)	<0.00001	−18.2 (−20.9; −15.5)	<0.00001	−9.9 (−13.1; −6.7)	<0.00001
SBQ					8.0 (4.9; 11.2)	<0.00001	−36.3 (−39.4; −33.2)	<0.00001	−28.0 (−31.5; −24.5)	<0.00001
COPD−SQ							−44.3 (−48.0; −41.1)	<0.00001	−36.0 (−39.6; −32.4)	<0.00001
PEF									8.3 (6.0; 10.6)	<0.00001

Values indicate the difference in specificity (with 95% CI & McNemar’s *p*-values), comparing the index test in the left column against index tests across the table.

The peak flow and microspirometry airflow measurement devices had lower sensitivity but significantly higher specificity compared to the questionnaires (Table 2). Peak flow meter had higher specificity 95.5% (95% CI 94.1%, 96.6%), while microspirometry had slightly higher sensitivity 50.6% (95% CI 39.1%, 62.1%).

Using combinations of two tests, requiring both to be positive for an overall positive result, had lower sensitivities but higher specificities than individual tests. Combinations including the SBQ or COPD-SQ questionnaire with either airflow measurement device provided similar specificity but improved sensitivity compared to using the other questionnaires. Combinations with peak flow had sensitivities of 39.2% (95% CI 28.4%, 50.0%) for both questionnaires, and specificities of 97.0% (95% CI 95.7%, 97.9%) and 96.7% (95% CI 95.4%, 97.7%) for SBQ and COPD-SQ, respectively. Combinations with microspirometry had similar sensitivities of 40.5% (95% CI 29.6%, 52.1%) and 41.8% (95% CI 30.8%, 53.4%) but slightly lower specificities of 93.7% (95% CI 92.1%, 95.1%) and 93.4% (95% CI 91.7%, 94.8%) for SBQ and COPD-SQ, respectively (Table 2). Overall, specificity estimates were higher when questionnaires were combined with peak flow rather than microspirometry, though sensitivity was similar regardless of the airflow device used.

### Economic evaluation of test combinations

After removal of dominated combinations that were more costly but less accurate (based on a combination of sensitivity and specificity), the most costly combination of screening tests was COPD-SQ and microspirometry, but this also detected the most true cases (Table 5). The incremental cost-effectiveness ratio (ICER) for COPD-SQ and microspirometry vs. the next best option of SBQ and microspirometry was £302 (R$992) per additional true case detected. The ICER for SBQ and peak flow (compared with CDQ and microspirometry) was much lower at £54 (R$178) per additional case detected. However, the optimal combination is dependent on the maximum willingness to pay in Brazil to detect an additional true case of COPD. Options including peak flow tended to be cheaper overall than those with microspirometry.

**Table 5:** Per patient cost, effectiveness and economic evaluation of selected test combinations.

Test combination	Cost per test combination (per 1000 patients) UK£ (R$)	Difference in cost^a^ (per 1000 patients) UK£ (R$)	True cases detected (per 1000 patients)	Difference in true cases detected (per 1000 patients)	ICER^a, b^ UK£ (R$) per additional true case detected
CDQ and Peak flow	1130 (3713)	—	17.2	—	—
CDQ and Microspirometry	1317 (4328)	187 (615)	19.8	2.6	72 (238)
SBQ and Peak flow	1690 (5554)	373 (1226)	26.7	6.9	54 (178)
SBQ and Microspirometry	2019 (6635)	329 (1081)	27.5	0.9	372 (1223)
COPD-SQ and Microspirometry	2286 (7512)	267 (877)	28.4	0.9	302 (992)

^a^Denotes difference between cost of the combination in this row, compared with the cost of the combination in the previous row.

^b^ICER: Incremental cost-effectiveness ratio.

The two combinations containing the CAPTURE questionnaire were both dominated (more costly, less effective) and are not shown.

## Discussion

We demonstrated that combining a simple questionnaire with an airflow measurement device was feasible to use as a screening tool for undiagnosed COPD amongst those with a known risk factor in the Brazilian primary care setting. In our sample, airflow measurement devices had higher specificity and questionnaires had higher sensitivity. The SBQ and COPD-SQ questionnaires had the highest sensitivity and reasonable specificity compared to the other questionnaires and peak flow had slightly better specificity and similar sensitivity compared to microspirometry. We found that using the SBQ or COPD-SQ with either of the airflow measurement devices would be an appropriate test combination, providing similar specificity but improved sensitivity compared to using the other questionnaires. The ease of use and cheaper unit cost of peak flow meters compared to microspirometers, as well as estimated performance when test combinations included peak flow, suggests that peak flow might be the better choice of airflow measurement device. With this in mind, SBQ and peak flow offer the preferred test combination, due to detecting only marginally fewer true cases and being cheaper than the combinations of SBQ or COPD-SQ with microspirometry. This combination also had the highest PPV out of all tests and combinations in our population. It is important to note, however, that PPV will vary based on the prevalence of COPD in different populations.

The sensitivity and specificity of the screening questionnaires in this study differed from those reported in validation studies for these instruments. These differences are likely to be explained by the difference in disease spectrum among the populations evaluated and by the reference standard used in our study. For example, the COPD-SQ^27^ and the CAPTURE^21^ validation study samples had 79% and 82% with a positive smoking history, respectively, compared to 51% in our sample. The CAPTURE study aimed to identify people with clinically significant COPD, defined as those with COPD and either a history of one or more exacerbations in the last year, or moderate to severe airflow obstruction (FEV1 < 60% predicted) and exacerbation-free for more than a year. This means that whilst our study may be more clinically useful in terms of implementation than the prior studies, because of multiple tools used and compared, comparison of individual test performance to other studies using a different approach may not be appropriate. In addition, the prevalence and spectrum of disease in the population under study can affect test performance, and our strategy to enrich the screened population will have affected this, and cannot be separated from the result.

Overall, the economic evaluation found test combinations including microspirometry to be the most effective but also the most expensive. Decisions regarding which combination to fund would require trade-offs between the number of true cases detected and the associated costs. The combinations of microspirometry and either COPD-SQ or SBQ detected the greatest number of true cases but were among the most expensive, whereas the combination of peak flow and SBQ detected slightly fewer true cases but was considerably cheaper. Decisions on the optimal strategy are also dependent on what the Brazilian health service is willing to pay to detect an additional case of COPD. Whilst thresholds are available when results are presented as cost per quality-adjusted life year (QALY), when the unit of effect is in ‘natural units’ such as cost per case detected, the threshold is unknown. Therefore, it is difficult to determine which is the most cost-effective strategy and whether R$8877 per additional case detected for microspirometry and COPD-SQ is value for money. Therefore, factors such as equipment cost and ease of use should also be taken into consideration.

In addition to differences in screening test performance based on characteristics of the underlying population, contextual factors such as availability and affordability of diagnostic services and treatment need to be considered. Within the current Brazilian context with limited resources for diagnostic spirometry, it is important to maximize screening test accuracy while prioritizing specificity, to minimize false positives and the number of people referred for diagnostic spirometry. In our study sample, we suggested that SBQ and peak flow might be the preferred combination of tests. Although the low sensitivity of 39.2% implied missing nearly two thirds of true cases, further analysis revealed those missed tended to be younger, never smokers, have no, or mild breathlessness and less severe airflow obstruction compared to those correctly detected by the screening test (Supplementary Table 1). Therefore, the proposed test combination identified those most likely to benefit from available effective treatments at the time of screening. Nevertheless, using this strategy would mean that some people with true COPD will be missed, a proportion of whom could have benefited from treatment. This may have implications for delayed treatment and later economic consequences.

Inclusion of the SBQ^28^ and the CDQ^22^ from which it was derived allowed direct comparison of both questionnaires. The removal of two items about productive cough and inclusion of five extra items about exposures, cough, breathlessness and childhood respiratory diseases, resulted in increased sensitivity and decreased specificity of the SBQ compared to the CDQ within the study sample.

The GOLD guideline recommends the use of a fixed ratio (FEV_1_/FVC < 0.7)^15^ to define COPD diagnosis, which would have altered the reported index test performances had we used this criteria. This is controversial and some publications question that using the fixed ratio will lead to overdiagnosis, particularly in patients over 65 years^29,30^. By using LLN in this study, those identified with airflow obstruction were more likely to represent true COPD, rather than an indication of other comorbidities such as cardiac disease^31^. The prevalence of COPD in our study was approximately half that found in São Paulo by the Platino study^10^ (7.8% vs. 15%), potentially due to the latter using FEV_1_/FVC < 0.7 to define obstruction and also not performing clinical evaluations on those identified.

A further advantage of our study is the confirmation of cases with a clinical assessment. In our view, the challenge in primary care is the imposition of diagnosis based only on lung function examination. Airflow obstruction alone cannot confirm a diagnosis of COPD, due to potential differential diagnoses such as severe asthma that may not respond to bronchodilation^32^. This differentiation has become increasingly important, because although asthma and COPD are obstructive diseases, they have pathophysiological and treatment differences^33^. Nevertheless, there is much debate around spirometric criteria and thresholds for bronchodilator response to differentiate between asthma and COPD, and changes in diagnostic criteria would affect our findings.

Identifying patients with COPD in primary care is still a challenge worldwide^26^. Finding feasible screening tests that can be applied in different parts of the world is essential, especially in low- and middle-income countries. We demonstrated that primary care clinicians can use simple tools for the investigation and screening of suspected obstructive lung disease, and then refer to a pulmonologist for clinical confirmation. This is an important finding, given the scarcity of medical specialists in Brazil^34^ and elsewhere. Spirometry is often unavailable or of poor quality in primary care settings, therefore screening with simple tests prior to specialist referral for confirmation, minimizes the risk of under or overdiagnosis due to errors.

This is one of the largest studies to examine the accuracy of different screening tests for undiagnosed COPD and one of the few conducted in a middle-income country, rather than a high-income setting where the tests were previously evaluated. Characteristics of people with undiagnosed COPD in high- and middle-settings may differ for various reasons, such as nutrition, lung growth and air pollution. For this study we did not exclude those who had a previous diagnosis of COPD. This may affect our findings if the pathophysiological characteristics of those with undiagnosed COPD differ from those who have previously diagnosed disease. However, we found that out of 114 who mentioned a previous diagnosis, only 29 were confirmed on assessment, suggesting high levels of inaccuracy for previous diagnosis.

Our results indicate that the combination of peak flow with either the SBQ or COPD-SQ was able to identify patients in primary care who warranted referral for diagnostic spirometry and clinical review. The decision model used in the clinical review was able to distinguish between patients with COPD, asthma-COPD overlap and asthma, thus enabling clinicians to treat patients appropriately. While the above test combination appears feasible, successful implementation of COPD screening programmes in Brazil would require clinicians having additional training to better recognize respiratory symptoms^35^, and additional resources to increase accessibility to screening tests and diagnostic spirometry.

## Methods

### Study design and population

We conducted a cross-sectional, screening test accuracy study to assess six different screening tests and their combinations for detecting COPD patients in Brazil. Study assessments were performed in nine basic health units, eight urban and one rural, in the city of São Bernardo do Campo, São Paulo, Brazil.

Between February and October 2019, eligible patients aged ≥40 years with clinician diagnosed hypertension who attended routine consultations at their registered Basic Health Unit were invited to attend a separate study assessment. Patients were excluded if they were unable to perform spirometry (dementia, lack of teeth, lack of coordination or not having a good oral seal), had contraindications for spirometry (respiratory infection, bloody cough in the last month, severe angina, systolic blood pressure ≥220 mmHg or diastolic blood pressure ≥120 mmHg), had a history of tuberculosis, cardiac infarction, retinal detachment or surgery on the chest, abdomen, brain, ears or eyes in the last 3 months, or had a prior adverse reaction to Salbutamol.

### Study assessment

Participants provided informed consent, prior to measurement of height and weight, and completion of all index and reference tests. They also completed a study questionnaire (Supplementary Methods 1) to provide information about demographics, smoking status, medical diagnoses, quality of life and respiratory symptoms. The questionnaire was developed in English, and translated to Portuguese by a professional translator, checked by Portuguese speaking clinicians to ensure the meaning of questions was preserved. For questionnaire items such as the CAPTURE questionnaire, where an existing validated Portuguese version was available, this was used. Case report forms were used to capture study assessment data and all data were entered on a secure online REDCap database^36,37^. We used a paired design, whereby all participants performed the index and reference tests during a single study assessment visit.

### Index tests

The index tests included peak flow (QVAR Mini Wright®, cut-point <350 l/min men, <250 l/min women)^21^, pre-bronchodilator microspirometry (Vitalograph-COPD6®, cut-point FEV_1_/FEV_6_ < 0.78)^38^, and four screening questionnaires, including COPD Diagnostic Questionnaire (CDQ, cut-point ≥20)^22,39^, CAPTURE (cut-point ≥ 2)^21^, COPD Screening Questionnaire (COPD-SQ, cut-point ≥ 16)^27^, and the symptom-based questionnaire (SBQ, cut-point ≥ 17)^28^. The selection of questionnaires maximized symptoms being assessed and minimized duplication of items, while allowing comparison of the most relevant questionnaires. The CDQ, CAPTURE, COPD-SQ, and SBQ items were included in the study questionnaire without repetition (Supplementary Methods 2); the full list of all four tools is available in Supplementary Methods 3.

Peak flow and microspirometry were conducted before questionnaires, with the order of the airflow measurement device alternating at each assessment to reduce learning bias. Trained researchers explained how to use both lung function tests, and participants performed three pre-bronchodilator maneuvers on each device. For each test, the best values from any of the three maneuvers were used for analysis.

Participants completed the questionnaires after receiving 400 micrograms of Salbutamol. Questionnaires were intended to be self-completed, but researchers could assist if required.

### Reference test

The reference test comprised post-bronchodilator quality diagnostic spirometry (ndd Easy On-PC) with clinical review to confirm COPD. Spirometry was administered by a second trained researcher who was unaware of the prior airflow measurement test results, between 20 and 60 min after bronchodilation, aiming for repeatability within 100 ml or 5%, within six efforts.

We assessed lung function with a spirometer that displayed and printed out the flow volume curve. The curves were classified according to the criteria of the ATS/ERS task force on standardization of lung function testing^40^. Tests with at least three curves, meeting these criteria, were “good.” “Usable” tests contained at least one curve that concurred with the criteria, allowing accurate assessment of FEV_1_. If accurate assessment was not possible the curves were classified as “unacceptable” and the test was excluded from analysis. All traces were over-read for quality by independent respiratory experts and graded according to standard criteria^40^, without knowledge of the index test results. Airflow obstruction was defined by the lower limit of normal (LLN) using Global Lung Initiative (GLI) equations.

A pulmonologist conducted clinical reviews with all participants whose diagnostic spirometry was below the LLN. If post-bronchodilator reversibility of FEV_1_ was ≥12%, and >400 mls patients were classed as having asthma, and were defined as reference test negative. Those with FEV_1_ reversibility ≥12% between 200 and 400 mls and a history of Asthma or allergies were classed as having asthma/COPD overlap. All others reviewed by the pulmonologist were classed as having COPD alone. The latter two groups were defined as reference test positive. These thresholds were based on local clinical guidelines in Brazil, and are in line with international diagnostic recommendations.

### Sample size

A pragmatic target of recruiting 120 patients per BHU was set, to obtain a total sample of approximately 1080 participants. Using the Alonzo method for paired test accuracy studies^41^, assuming independence of tests and a prevalence of 16%, we would have 85% power to detect a difference in sensitivity of 10% (95% vs. 85%) with 1040 participants. If the sensitivity of tests was slightly lower in this population (91% vs. 80%) we would have 80% power to detect this difference with the same sample size.

### Statistical analysis

The diagnostic performance of each index test was investigated by presenting 2 × 2 tables and calculating the sensitivity, specificity, positive predictive value and negative predictive value with 95% confidence intervals. Comparative test accuracy was assessed by calculating the difference in sensitivity and specificity, presenting 95% confidence intervals and using McNemar’s test.

The primary analysis compared the sensitivity and specificity between the CAPTURE screening questionnaire and peak flow meter, as this combination has been previously developed to be more relevant for low-resource settings. Secondary analyses compared the comparative performance of all other individual index tests, as well as likely test combinations. The test combinations aimed to maximize specificity (positive result on both index tests), thus in future optimizing efficiency by limiting the number of people requiring diagnostic spirometry. Where it was necessary to for our health economic analyses, test accuracy assessment was based on balancing test sensitivity and specificity, although within the context of the low-resource setting in Brazil, specificity was prioritized where the balance was not clear cut. All analyses were conducted in Stata v16 (Windows Stata—Stata Corp LLC™)

### Economic analysis

We conducted a cost-effectiveness analysis to calculate the cost per additional true case detected. The combinations were ordered by the number of true cases detected, from least to greatest, and the principle of dominance applied to eliminate redundant combinations from the analysis (where they were more costly and less effective). Each test was then compared with the next best alternative. For the purpose of this paper, we compared test combinations, rather than individual screening tests.

The unit costs and quantity of any equipment, medication and consumables required, staff time (and salary costs) to deliver each individual test and use of facilities were determined to calculate the health-care costs of delivering each screening combination. Each individual test was timed at a sample of assessment clinics to estimate an overall mean time and range for each test. Equipment costs were depreciated (at 3.5% a year) over the estimated lifespan of the equipment (ranging from 1 to 5 years). Cost per patient visit was calculated assuming the equipment would be used for 750 patients per clinic per year. It was also assumed that true and false positive cases would require GP reassessment, confirmation with quality diagnostic spirometry (assuming 1,000 patients/year) and a clinical review with a pulmonologist (for true positives). Costs were calculated in UK£ for a price year of 2019 and converted to Brazilian Real (R$) using Purchasing Power Parities (PPP)^42^ with a conversion rate of 3.29 (Supplementary Methods 4).

The paper was written according to the STARD guidance^43^ for reporting studies of diagnostic accuracy.

### Ethics

This study was carried out in accordance with good clinical practices and was approved by the Ethics Committees of the ABC Medical School, Sao Paulo, Brazil on February 4, 2019 (no. 3.131.048) and the University of Birmingham, Birmingham, UK (ERN_18-1185).

### Reporting summary

Further information on research design is available in the Nature Research Reporting Summary linked to this article.

## Supplementary information

Supplementary data

REPORTING SUMMARY

## Data Availability

Data are available upon reasonable request. All data requests should be submitted to author PA for consideration. Access to anonymised data may be granted following review.

## References

[1] Lozano, R. et al. Global and regional mortality from 235 causes of death for 20 age groups in 1990 and 2010: a systematic analysis for the Global Burden of Disease Study 2010. *Lancet* **380**, 2095–2128 (2012). 10.1016/S0140-6736(12)61728-0 PMC10790329 23245604

[2] European Respiratory Society. In *Respiratory Health and Disease in Europe* (eds. Gibson, G. J., Loddenkemper, R., Sibille, Y. & Lundback, B.) (European Respiratory Society, 2013). 10.1183/09031936.00105513 24000245

[3] Rehman AU (2020). The economic burden of chronic obstructive pulmonary disease (COPD) in Europe: results from a systematic review of the literature. Eur. J. Health Econ..

[4] National Institute for Health and Care Excellence. *Chronic Obstructive Pulmonary Disease: Management of Chronic Obstructive Pulmonary Disease in Adults in Primary and Secondary Care* (National Institute for Health and Care Excellence, 2010).

[5] Roche N (2010). Acute respiratory illness as a trigger for detecting chronic bronchitis in adults at risk of COPD: a primary care survey. Prim. Care Respiratory J.: J. Gen. Pract. Airw. Group.

[6] Ho T, Cusack RP, Chaudhary N, Satia I, Kurmi OP (2019). Under- and over-diagnosis of COPD: a global perspective. Breathe.

[7] Langsetmo L, Platt RW, Ernst P, Bourbeau J (2008). Underreporting exacerbation of chronic obstructive pulmonary disease in a longitudinal cohort. Am. J. Respir. Crit. Care Med.

[8] Lamprecht B (2015). Determinants of underdiagnosis of COPD in national and international surveys. Chest.

[9] Hangaard S, Helle T, Nielsen C, Hejlesen OK (2017). Causes of misdiagnosis of chronic obstructive pulmonary disease: a systematic scoping review. Respir. Med.

[10] Menezes AM (2005). Chronic obstructive pulmonary disease in five Latin American cities (the PLATINO study): a prevalence study. Lancet.

[11] Queiroz MC, Moreira MA, Rabahi MF (2012). Underdiagnosis of COPD at primary health care clinics in the city of Aparecida de Goiania, Brazil. J. Bras. Pneumol..

[12] Diab N (2018). Underdiagnosis and overdiagnosis of chronic obstructive pulmonary disease. Am. J. Respir. Crit. Care Med..

[13] Preteroti, M. et al. Population-based case-finding to identify subjects with undiagnosed asthma or COPD. *Eur. Respir. J.* **55**, 2000024 (2020). 10.1183/13993003.00024-2020 32299864

[14] National Institute for Health and Care Excellence. *Chronic Obstructive Pulmonary Disease in Over 16s: Diagnosis and Management* (National Institute for Health and Care Excellence, 2018). 31211541

[15] Global Initiative for Chronic Obstructive Lung Disease. *Global Strategy for the Diagnosis, Management, and Prevention of Chronic Obstructive Pulmonary Disease (2019 report)* (Global Initiative for Chronic Obstructive Lung Disease, 2019).

[16] Talamo C (2007). Diagnostic labeling of COPD in five Latin American cities. Chest.

[17] US Preventive Services Task Force. Screening for chronic obstructive pulmonary disease: US Preventive Services Task Force recommendation statement. *JAMA* **315**, 1372–1377 (2016).

[18] UK National Screening Committee. *An Evaluation of Screening for COPD Against the National Screening Committee Criteria* (UK National Screening Committee, 2013).

[19] Laucho-Contreras, M. E. & Cohen-Todd, M. Early diagnosis of COPD: myth or a true perspective. *Eur. Respir. Rev.* **29**, 200131 (2020). 10.1183/16000617.0131-2020 PMC9489086 33268437

[20] Lambe T (2019). Model-based evaluation of the long-term cost-effectiveness of systematic case-finding for COPD in primary care. Thorax.

[21] Martinez FJ (2017). A new approach for identifying patients with undiagnosed chronic obstructive pulmonary disease. Am. J. Respir. Crit. Care Med..

[22] Price DB (2006). Symptom-based questionnaire for identifying COPD in smokers. Respiration.

[23] Frith P (2011). Simplified COPD screening: validation of the PiKo-6 in primary care. Prim. Care Respir. J..

[24] Represas-Represas C (2016). Screening for chronic obstructive pulmonary disease: validity and reliability of a portable device in non-specialized healthcare settings. PLoS ONE.

[25] Ronaldson SJ (2018). Determining the optimal approach to identifying individuals with chronic obstructive pulmonary disease: the DOC study. J. Eval. Clin. Pr..

[26] Haroon S, Jordan R, Takwoingi Y, Adab P (2015). Diagnostic accuracy of screening tests for COPD: a systematic review and meta-analysis. BMJ Open.

[27] Zhou YM (2013). Development and validation of a chronic obstructive pulmonary disease screening questionnaire in China. Int. J. Tuberc. Lung Dis..

[28] Zhang Q, Wang M, Li X, Wang H, Wang J (2016). Do symptom-based questions help screen COPD among Chinese populations?. Sci. Rep..

[29] Garcia-Rio F (2011). Overdiagnosing subjects with COPD using the 0.7 fixed ratio: correlation with a poor health-related quality of life. Chest.

[30] Kainu, A., Timonen, K., Lindqvist, A. & Piirila, P. GOLD criteria overestimate airflow limitation in one-third of cases in the general Finnish population. *ERJ Open Res*. **2**, 00084-2015 (2016). 10.1183/23120541.00084-2015 PMC5152847 28053971

[31] van Dijk W (2015). Clinical relevance of fixed ratio vs lower limit of normal of FEV1/FVC in COPD: patient-reported outcomes from the CanCOLD cohort. Ann. Fam. Med..

[32] Rogliani P, Ora J, Puxeddu E, Cazzola M (2016). Airflow obstruction: is it asthma or is it COPD?. Int. J. Chronic Obstr. Pulm. Dis..

[33] Global Initiative for Asthma (GINA). *Global Strategy for Asthma Management and Prevention* (Global Initiative for Asthma, 2021).

[34] Scheffer, M. et al. *Demografia Medica no Brasil 2018*. 286p. (FMUSP, CFM, Cremesp, Sao Paulo, SP, 2018).

[35] Martins SM (2016). Implementation of ‘matrix support’ (collaborative care) to reduce asthma and COPD referrals and improve primary care management in Brazil: a pilot observational study. NPJ Prim. Care Respir. Med..

[36] Harris PA (2009). Research electronic data capture (REDCap)-a metadata-driven methodology and workflow process for providing translational research informatics support. J. Biomed. Inf..

[37] Harris PA (2019). The REDCap consortium: building an international community of software platform partners. J. Biomed. Inf..

[38] Labor M, Vrbica Z, Gudelj I, Labor S, Plavec D (2016). Diagnostic accuracy of a pocket screening spirometer in diagnosing chronic obstructive pulmonary disease in general practice: a cross sectional validation study using tertiary care as a reference. BMC Fam. Pract..

[39] Stanley AJ, Hasan I, Crockett AJ, van Schayck OC, Zwar NA (2014). COPD Diagnostic Questionnaire (CDQ) for selecting at-risk patients for spirometry: a cross-sectional study in Australian general practice. NPJ Prim. Care Respir. Med.

[40] Miller MR (2005). ATS/ERS task force: standardisation of spirometry. Eur. Respir. J..

[41] Alonzo TA, Pepe MS, Moskowitz CS (2002). Sample size calculations for comparative studies of medical tests for detecting presence of disease. Stat. Med..

[42] OECD. *Purchasing Power Parities (PPP) (indicator)* (OECD, 2020).

[43] Bossuyt PM (2015). STARD 2015: an updated list of essential items for reporting diagnostic accuracy studies. BMJ.

