# Supplementary material for: Accuracy and economic evaluation of screening tests for undiagnosed COPD among hypertensive individuals in Brazil
Source: NPJ Prim Care Respir Med. 2022 Dec 13;32:55. doi: 10.1038/s41533-022-00303-w (PMC9747958; doi:10.1038/s41533-022-00303-w)
Supplement: Supplementary file 1 — Supplementary data [file 41533_2022_303_MOESM1_ESM.docx]

Supplementary Table 1: Comparison of true COPD cases detected and missed by the SBQ and peak flow test combination

|  | **True positives (n=31)** | **False Negatives (n=48)** |
| --- | --- | --- |
| Sex; n (%) male | 20 (64.5) | 19 (39.6) |
| Age; mean (SD) | 70.9 (8.0) | 63.6 (11.6) |
| mMRC; n (%)  Grade 0-1  Grade 2-4 | 18 (58.1)  13 (41.9) | 37 (77.1)  11 (22.9) |
| Smoking status; n (%)  Current  Ex  Never | 4 (12.9)  25 (80.7)  2 (6.5) | 17 (35.4)  23 (47.9)  8 (16.7) |
| GOLD stage; n (%)  I (FEV1 ≥80% predicted)  II (FEV1 50-79% predicted)  III (FEV1 30-49% predicted)  IV (FEV1 <30% predicted) | 2 (6.5)  18 (58.1)  10 (32.3)  1 (3.2) | 17 (35.4)  27 (56.3)  4 (8.3)  - |

Supplementary Methods 1: Study questionnaire

**Evaluating screening strategies for identifying undiagnosed COPD in Brazil: a Breathe Well project**

Study Questionnaire

| Patient Initials |  |
| --- | --- |
| Study ID |  |
| Date |  |
| Interviewer ID |  |

Your answers and opinions are valuable to us. We would be very grateful if you could read the below before turning the page:

- Please complete this questionnaire yourself if at all possible
- Please answer all questions as well as you can
- Do not spend too long thinking about your answers
- If someone is completing this on your behalf, they should record your answers


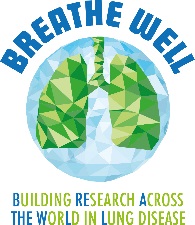


**1. What is your age?** ______ years old

2. **Gender**: □Female □Male

3. **Nationality**: **□**Brazil **□** Foreigner **□** Naturalized

**4. Race: □**White **□** Yellow **□** Black **□**Red (Indian) **□**Another

5. **Marital status**:

**□**Single **□** Married **□**Widow(er) **□**Separated (not officially) **□**Separated (old Brazilian law) **□**Divorced **□** Cohabiting

**6. What is the highest level of qualification that you have?**

|  | Unlettered |
| --- | --- |
|  | Elementary school I incomplete |
|  | Elementary school I complete |
|  | Elementary school II incomplete |
|  | Elementary school II complete |
|  | High school incomplete |
|  | High school complete |
|  | Bacharel |
|  | Master |
|  | Doctor |

**7. What do you do?**

**□**Work and study

**□** Work only

**□** On leave or incapacitated to work

**□** Retired

**□** Neither working nor studying

**8. What is your profession or main occupation? ______________________**

**9. Where have you spent most of your life?**

**□**Urban area

**□**Rural Area

**10. Has a doctor EVER told you that you had any of the following conditions? Please tick all that apply?**

|  | Yes | No |
| --- | --- | --- |
| Chronic Obstructive Pulmonary Disease |  |  |
| Chronic bronchitis/emphysema |  |  |
| Asthma |  |  |
| Tuberculosis |  |  |
| Diabetes Mellitus |  |  |
| Anxiety |  |  |
| Depression |  |  |
| Heart disease |  |  |
| Cancer |  |  |

**11. What is your current smoking status?**

**□**Current smoker smoker (smoke at least 1 cigarette per day for at least the last 6 months)

**□**Ex smoker (previously smoked at least 1 cigarette per day for at least 6 months, but not now)

**□**Never smoker (please go to Q14)

**12. If you have ever smoked, at about what age did you start to smoke regularly? (by regularly we mean at least 1 cigarette/day or 7 cigarettes/week for at least 6 months)**

_______ years old

If you are an ex-smoker, at what age did you **stop** smoking regularly?

_______ years old

**13．How much do you usually smoke each day now, or did you usually smoke when you were a smoker?**

| Filter cigarettes |  | number/day |
| --- | --- | --- |
| Non-filter/hand rolled cigarettes |  | number/day |
| Straw cigarettes |  | number/day |
| Cigars (including as part of your religion) |  | number/day |
| Water pipe |  | g/day tobacco |
| Electronic cigarettes (or e-cigarettes) |  | number/day |

14. Which of the following chemicals or particulates are you currently exposed to at work/home, or which have you been exposed to at work/home in the past? (Biomass fuel consists of fire wood, manure, agricultural crop residues such as straw/grass/shrubs, coal fuels and kerosene)

| Chemicals or particulates | Yes, currently | Yes, in the past | No, never |
| --- | --- | --- | --- |
| Cooking fumes |  |  |  |
| Biomass fuel |  |  |  |
| Steam of various substances |  |  |  |
| Gas |  |  |  |
| Dust |  |  |  |

15. If you ticked ‘yes’ to any exposures, how many years have you been exposed to them?

______years

16. If exposed to cooking fumes or biomass fuels, did the home/workplace have a chimney or exhaust system?

Yes

No

Please complete the below questionnaire even if you do not have a lung condition

**17. How are your lung problems? For each item below circle the number that best describes your experience on a scale of 0-5**


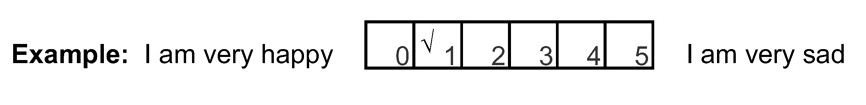


| **I never cough** | **0 1 2 3 4 5** | **I cough all the time** |
| --- | --- | --- |
| **I have no phlegm (mucus) in my chest at all** | 0 1 2 3 4 5 | My chest is completely full of phlegm (mucus) |
| **My chest does not feel tight at all** | 0 1 2 3 4 5 | My chest feels very tight |
| **When I walk up a hill or one flight of stairs I am not breathless** | 0 1 2 3 4 5 | When I walk up a hill or one flight of stairs I am very breathless |
| **I am not limited doing any activities at home** | 0 1 2 3 4 5 | I am very limited doing activities at home |
| **I am confident leaving my home despite my lung condition** | 0 1 2 3 4 5 | I am not at all confident leaving my home because of my lung condition |
| **I sleep soundly** | 0 1 2 3 4 5 | I don’t sleep soundly because of my lung condition |
| **I have lots of energy** | 0 1 2 3 4 5 | I have no energy at all |

COPD Assessment test and the CAT logo is a trade mark of the GlaxoSMithKline group of companies. ©2009 GlaxoSmithKline group of companies. All rights reserved.

**18. Please circle the best response to describe your shortness of breathe:**

| Grade | Description of breathlessness |
| --- | --- |
| **0** | I only become breathless with strenuous exercise |
| **1** | I get short of breath when hurrying on the level or walking up a slight hill |
| **2** | I walk slower than most people the same age on the level because of breathlessness, or I have to stop for breath when walking at my own pace on the level |
| **3** | I stop for breath after walking about 100 metres or after a few minutes on the level |
| **4** | I am too breathless to leave the house or I am breathless when dressing or undressing |

Supplementary Methods 2: Lung health questionnaire

**
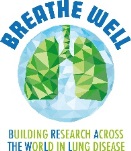
**

**Evaluating screening strategies for identifying undiagnosed COPD amongst hypertensive patients in Brazil: a Breathe Well project**

**Lung health questionnaire**

| Participant Initials |  |
| --- | --- |
| Study ID |  |
| Date |  |
| Interviewer ID |  |

Some questions in the following booklets may appear similar. However, it is important that we ask these questions in slightly different ways so please complete all questions, answering them as accurately as possible.

**CDQ**

1. Age group, years

| 40–49 | 50-59 | 60-69 | 70+ |
| --- | --- | --- | --- |

1. What is your weight in kilograms?

| <25.4 | 25.4–29.7 | >29.7 |
| --- | --- | --- |

What is your height in meters?

_____________ metres

1. Smoking

What is the total number of years you have smoked?

_____ years

How many cigarettes do you currently smoke each day (or ‘did smoke each day’ if ex-smoker)?

_____ cigarettes

1. Does the weather affect your cough?

| Yes | No |
| --- | --- |

1. Do you ever cough up phlegm (sputum) from your chest when you don’t have a cold?

| Yes | No |
| --- | --- |

1. Do you usually cough up phlegm (sputum) from your chest first thing in the morning?

| Yes | No |
| --- | --- |

1. How frequently do you wheeze?

| Occasionally or more often | Never |
| --- | --- |
|  |  |

1. Do you have or have you had any allergies?

| Yes | No |
| --- | --- |

**CAPTURE**

1. Have you ever lived or worked in a place with dirty or polluted water or air, smoke or second-hand smoke or dust?

| Yes | No |
| --- | --- |

1. Does your breathing change with seasons, weather or air quality?

| Yes | No |
| --- | --- |

1. Does your breathing make it difficult to do things such as carry heavy loads, shovel dirt or snow, jog, play tennis or swim?

| Yes | No |
| --- | --- |

1. Compared to others your age, do you tire easily?

| Yes | No |
| --- | --- |

1. In the past 12 months, how many times did you miss work, school, or other activities due to a cold, bronchitis, or pneumonia?

| 0 | 1 | 2 or more |
| --- | --- | --- |

Copyright© 2015 by Cornell University, University of Kentucky, and Evidera. All Rights Reserved

**Symptom-based questionnaire**

1. How frequently are you exposed to second-hand smoking?

| <7hrs per week | ≥7hrs per week |
| --- | --- |

1. Do you often cough when you do not have a cold?

| Yes | No |
| --- | --- |

1. Do you have more signs of shortness of breath compared with others of the same age?

| Yes | No |
| --- | --- |

1. Have you had long-term exposure to dust or chemical particles?

| Yes | No |
| --- | --- |

1. Did you have a history of chronic respiratory diseases when you were a child?

| Yes | No |
| --- | --- |

**COPD-SQ**

1. Do you often cough?

| Yes | No |
| --- | --- |

1. Family history of respiratory disease

| Yes | No |
| --- | --- |

1. Exposure to biomass smoke from cooking fires

| Yes | No |
| --- | --- |

Supplementary Methods 3: Screening questionnaires used in the study

| **Questionnaire** | **Questions** | **Response categories** |
| --- | --- | --- |
| CAPTURE^21^ | 1. Have you ever lived or worked in a place with dirty or polluted water or air, smoke or second-hand smoke or dust? | Yes/no |
|  | 1. Does your breathing change with seasons, weather or air quality? | Yes/no |
|  | 1. Does your breathing make it difficult to do things such as carry heavy loads, shovel dirt or snow, jog, play tennis or swim? | Yes/no |
|  | 1. Compared to others your age, do you tire easily? | Yes/no |
|  | 1. In the past 12 months, how many times did you miss work, school, or other activities due to a cold, bronchitis, or pneumonia? | 0 / 1 / 2 or more |

| COPD Diagnostic Questionnaire (CDQ)^22^ | 1. What is your age in years? | 40–49 / 50–59 / 60–69 / 70+ |
| --- | --- | --- |
|  | 1. How many cigarettes do you currently smoke each day (or ‘did smoke each day’ if ex-smoker)?   What is the total number of years you have smoked?  Packs per day = cigarettes per day/20 per pack  Pack-years = packs per day × years smoked | 0–14 / 15–24 / 25–49 / 50+ |
|  | 1. What is your weight in kilograms?   What is your height in meters?  Body mass index (BMI) = weight (kg) / (height (m))^2^ | <25.4 / 25.4–29.7 / >29.7 |
|  | 1. Does the weather affect your cough? | Yes/no |
|  | 1. Do you ever cough up phlegm (sputum) from your chest when you don’t have a cold? | Yes/no |
|  | 1. Do you usually cough up phlegm (sputum) from your chest first thing in the morning? | Yes/no |
|  | 1. How frequently do you wheeze? | Occasionally or more often / never |
|  | 1. Do you have or have you had any allergies? | Yes/no |

| COPD-SQ^27^ | 1. How old are you? | 40–49 / 50–59 / 60–69 / 70+ |
| --- | --- | --- |
|  | 1. Do you often cough? | Yes/no |
|  | 1. Body mass index (BMI) = weight (kg) / (height (m))^2^ | <18.5 / 18.5–23.9 / 24.0–27.9 / ⩾28.0 |
|  | 1. Smoking intensity (average number of packs of cigarettes   smoked per day multiplied by smoking years) | Never smoker / 1–14.9 / 15–29.9 / ⩾30 pack-years |
|  | 1. Family history of respiratory disease | Yes/no |
|  | 1. Exposure to biomass smoke from cooking fires | Yes/no |
|  | 1. Which is the best description for your dyspnoea? | MRC scale 1-5 |

| Symptom based questionnaire (SBQ)^28^ | 1. What is your age in years? | 40–49 / 50–59 / 60–69 / 70+ |
| --- | --- | --- |
|  | 1. How many cigarettes do you currently smoke each day (if you are an ex-smoker, how many did you smoke each day)?   What is the total number of years you have smoked cigarettes?  Packs per day = cigarettes per day/20 per pack  Pack-years = packs per day × years smoked | 0–14 / 15–24 / 25–49 / 50+ |
|  | 1. What is your weight in kilograms?   What is your height in meters?  Body mass index (BMI) = weight (kg) / (height (m))^2^ | <24 / 24-28 / 28+ |
|  | 1. Does weather affect your cough? | Yes/no |
|  | 1. How frequently do you wheeze? | Occasionally or more often / never |
|  | 1. Do you have or have you had any allergies? | Yes/no |
|  | 1. How frequently are you exposed to second-hand smoking? | <7hrs per week / ≥7hrs per week |
|  | 1. Do you often cough when you do not have a cold? | Yes/no |
|  | 1. Do you have more signs of shortness of breath compared with others of the same age? | Yes/no |
|  | 1. Have you had long-term exposure to dust or chemical particles? | Yes/no |
|  | 1. Did you have a history of chronic respiratory diseases when you were a child? | Yes/no |

Supplementary Methods 4: Costs, timings and assumptions for screening strategies

| **Assessment timings** | **Minutes per patient** |
| --- | --- |
| Questionnaires (completion and processing) | 12 |
| Peak flow | 1.5 |
| Microspirometry | 2.5 |
| Confirmatory NDD spirometry | 6 |
| **Staff** | **Hourly costs (UK £)** |
| Nursing technician | 2.78 |
| GP | 14.03 |
| Pulmonologist | 13.53 |
| **Additional unit costs** | **(UK £)** |
| ***Questionnaire*** | 0.10 |
| ***Peak flow*** |  |
| Mouthpiece cost per patient | 2.00 |
| Overall equipment cost | 20.00 |
| Other consumable costs per patient | 0.21 |
| ***Microspirometry (COPD-6)*** |  |
| Mouthpiece cost per patient | 2.00 |
| Overall equipment cost | 100 |
| Battery cost per year | 20 |
| Other consumable costs per patient | 0.21 |
| ***Confirmatory NDD spirometry*** |  |
| Mouthpiece cost per patient | 4.00 |
| Overall equipment cost | 1,500 |
| Salbutamol cost per patient | 0.07 |
| Other consumable and equipment costs per patient | 0.66 |
| **Assumptions** |  |
| Number of visits per year per case finding clinic (assuming 15 patients per week, 50 weeks a year) | 750 |
| Number of visits per year per NDD spirometry clinic (assuming 20 patients per week, 50 weeks a year) | 1,000 |
| Lifetime of peak flow meter | 2 years |
| Lifetime of microspirometry | 2 years |
| Lifetime of NDD spirometry | 5 years |
| Proportion of patients requiring staff assistance with questionnaire | 98 |
| **Cost of case finding method per patient** | **(UK £)** |
| All questionnaires (CAPTURE, CDQ, SBQ, COPD-SQ) | 0.34 |
| Peak flow | 2.30 |
| Microspirometry | 2.43 |
| **Cost of confirmation method per patient** | **(UK £)** |
| GP reassessment before NDD (10 minute appointment) | 2.34 |
| Confirmatory NDD spirometry | 5.30 |
| Clinical review pulmonologist (10 minute appointment) | 2.26 |
