# Supplementary material for: Accuracy and economic evaluation of screening tests for undiagnosed COPD among hypertensive individuals in Brazil
Source: NPJ Prim Care Respir Med. 2022 Dec 13;32:55. doi: 10.1038/s41533-022-00303-w (PMC9747958; doi:10.1038/s41533-022-00303-w)
Supplement: Supplementary file 2 — REPORTING SUMMARY [file 41533_2022_303_MOESM2_ESM.pdf]

nature portfolio

Corresponding author(s): Rachel Adams, Rachel Jordan

Last updated by author(s): Sep 8, 2022

Reporting Summary

Nature Portfolio wishes to improve the reproducibility of the work that we publish. This form provides structure for consistency and transparency in reporting. For further information on Nature Portfolio policies, see our Editorial Policies and the Editorial Policy Checklist.

Statistics

For all statistical analyses, confirm that the following items are present in the figure legend, table legend, main text, or Methods section.

n/a	Confirmed	
☐	☒	The exact sample size (*n*) for each experimental group/condition, given as a discrete number and unit of measurement
☐	☒	A statement on whether measurements were taken from distinct samples or whether the same sample was measured repeatedly
☐	☒	The statistical test(s) used AND whether they are one- or two-sided<br>*Only common tests should be described solely by name; describe more complex techniques in the Methods section.*
☐	☒	A description of all covariates tested
☒	☐	A description of any assumptions or corrections, such as tests of normality and adjustment for multiple comparisons
☐	☒	A full description of the statistical parameters including central tendency (e.g. means) or other basic estimates (e.g. regression coefficient) AND variation (e.g. standard deviation) or associated estimates of uncertainty (e.g. confidence intervals)
☒	☐	For null hypothesis testing, the test statistic (e.g. *F*, *t*, *r*) with confidence intervals, effect sizes, degrees of freedom and *P* value noted<br>*Give P values as exact values whenever suitable.*
☒	☐	For Bayesian analysis, information on the choice of priors and Markov chain Monte Carlo settings
☒	☐	For hierarchical and complex designs, identification of the appropriate level for tests and full reporting of outcomes
☐	☒	Estimates of effect sizes (e.g. Cohen's *d*, Pearson's *r*), indicating how they were calculated

Our web collection on statistics for biologists contains articles on many of the points above.

Software and code

Policy information about availability of computer code

Data collection	n/a
Data analysis	n/a

For manuscripts utilizing custom algorithms or software that are central to the research but not yet described in published literature, software must be made available to editors and reviewers. We strongly encourage code deposition in a community repository (e.g. GitHub). See the Nature Portfolio guidelines for submitting code & software for further information.

Data

Policy information about availability of data

All manuscripts must include a data availability statement. This statement should provide the following information, where applicable:

- Accession codes, unique identifiers, or web links for publicly available datasets
- A description of any restrictions on data availability
- For clinical datasets or third party data, please ensure that the statement adheres to our policy

Data are available upon reasonable request. All data requests should be submitted to author PA for consideration. Access to anonymised data may be granted following review.

Human research participants

Policy information about studies involving human research participants and Sex and Gender in Research.

Reporting on sex and gender	When collecting data on characteristics of study participants, they were asked about their binary biological sex, but no questions were asked about gender identity.<br>Sex; n (%) male = 339 (31.3)
Population characteristics	Sex; n (%) male = 339 (31.3)
Recruitment	Between February and October 2019, eligible patients aged ≥40 years with clinician diagnosed hypertension who attended routine consultations at their registered Basic Health Unit were invited to attend a separate study assessment
Ethics oversight	Ethics Committees of the ABC Medical School, Sao Paulo, Brazil on February 4, 2019 (no. 3.131.048) and the University of Birmingham, Birmingham, UK (ERN_18-1185).

Note that full information on the approval of the study protocol must also be provided in the manuscript.

Field-specific reporting

Please select the one below that is the best fit for your research. If you are not sure, read the appropriate sections before making your selection.

☐ Life sciences ☒ Behavioural & social sciences ☐ Ecological, evolutionary & environmental sciences

For a reference copy of the document with all sections, see nature.com/documents/nr-reporting-summary-flat.pdf

Behavioural & social sciences study design

All studies must disclose on these points even when the disclosure is negative.

Study description	quantitative cross-sectional study
Research sample	Patients aged ≥40 years with clinician diagnosed hypertension who attended routine consultations at their registered Basic Health Unit, in Brazil
Sampling strategy	Between February and October 2019, eligible patients aged ≥40 years with clinician diagnosed hypertension who attended routine consultations at their registered Basic Health Unit were invited to attend a separate study assessment. Patients were excluded if they were unable to perform spirometry (dementia, lack of teeth, lack of coordination or not having a good oral seal), had contraindications for spirometry (respiratory infection, bloody cough in the last month, severe angina, systolic blood pressure ≥220mmHg or diastolic blood pressure ≥120mmHg), had a history of tuberculosis, cardiac infarction, retinal detachment or surgery on the chest, abdomen, brain, ears or eyes in the last three months, or had a prior adverse reaction to Salbutamol.
Data collection	Various data collection tools were used, including: study questionnaires, and REDCap database, peak flow, pre-bronchodilator microspirometry, symptom-based questionnaires, post-bronchodilator spirometry.
Timing	February to October 2019
Data exclusions	If accurate assessment was not possible the curves were classified as “unacceptable” and the test was excluded from analysis. All traces were over-read for quality by independent respiratory experts and graded according to standard criteria40, without knowledge of the index test results.<br>Withdrew during study (n=16)<br>Contraindicated for spirometry (n=15)<br>Unusable spirometry (n=25)<br>Incomplete study data (n=14)
Non-participation	Withdrew during study (n=16)<br>Contraindicated for spirometry (n=15)
Randomization	n/a

Reporting for specific materials, systems and methods

We require information from authors about some types of materials, experimental systems and methods used in many studies. Here, indicate whether each material, system or method listed is relevant to your study. If you are not sure if a list item applies to your research, read the appropriate section before selecting a response.

Materials & experimental systems

n/a	Involved in the study
☒	☐ Antibodies
☒	☐ Eukaryotic cell lines
☒	☐ Palaeontology and archaeology
☒	☐ Animals and other organisms
☐	☒ Clinical data
☒	☐ Dual use research of concern

Methods

n/a	Involved in the study
☒	☐ ChIP-seq
☒	☐ Flow cytometry
☒	☐ MRI-based neuroimaging

Clinical data

Policy information about clinical studies

All manuscripts should comply with the ICMJE guidelines for publication of clinical research and a completed CONSORT checklist must be included with all submissions.

Clinical trial registration	ISRCTN registration number: 11377960<br>Ethics Committees of the ABC Medical School, Sao Paulo, Brazil on February 4, 2019 (no. 3.131.048) and the University of Birmingham, Birmingham, UK (ERN_18-1185)
Study protocol	Protocols are available upon reasonable request from author PA.
Data collection	Study assessments were performed in nine basic health units, eight urban and one rural, in the city of São Bernardo do Campo, São Paulo, Brazil.
Outcomes	to assess the accuracy and associated costs of selected individual screening tests and their combinations for detecting undiagnosed COPD in a primary care setting. The reference test was defined as those below the lower limit of normal on quality diagnostic spirometry, with confirmed COPD at clinical review.
